# Running therapy improves clinical symptoms but not functional network connectivity in individuals with affective disorders

**DOI:** 10.1016/j.nicl.2025.103812

**Published:** 2025-05-27

**Authors:** Chris Vriend, Josine E. Verhoeven, Laura S. van Velzen, Lianne Schmaal, Brenda W.J.H. Penninx, Laura K.M. Han

**Affiliations:** a Amsterdam UMC Location Vrije Universiteit Amsterdam, Psychiatry, De Boelelaan 1117, Amsterdam, the Netherlands; b Amsterdam UMC Location Vrije Universiteit Amsterdam, Anatomy and Neurosciences, De Boelelaan 1117, Amsterdam, the Netherlands; c Amsterdam Neuroscience, Brain Imaging, Amsterdam, the Netherlands; d Centre for Youth Mental Health, The University of Melbourne, Parkville, VIC, Australia; e Orygen, Parkville, VIC, Australia; f Amsterdam Neuroscience, Mood, Anxiety, Psychosis, Sleep & Stress Program, Amsterdam, the Netherlands

**Keywords:** Exercise, Intervention, Trial, Psychiatry, rsfMRI, Affective disorders

## Abstract

**Objectives:**

Running therapy has been shown to be efficacious for depression and anxiety disorders. However, little is known about the effects of running therapy on functional brain connectivity.

**Methods:**

We used resting-state functional MRI scans from 25 individuals with an affective disorder that performed 16 weeks running therapy as part of the MOod Treatment with Antidepressants or Running (MOTAR) study. Using an atlas-based approach we investigated therapy-induced changes in connectivity and topology of several functional systems, e.g. the default-mode and salience networks. We additionally performed a case-control analysis using the pre-treatment scans of affective disorder patients (N = 50) and matched healthy controls (N = 66).

**Results:**

Running therapy significantly improved depressive (Inventory of Depressive Symptoms; IDS) and anxiety (Beck’s Anxiety Inventory; BAI) symptoms after 16 weeks (IDS: Z = −4.13, P < 0.001, BAI: Z = −2.87, P = 0.003), but it had no significant effect on functional connectivity or network topology. The case-control analyses at baseline also did not reveal any between-group differences.

**Conclusion:**

We conclude that there were either no functional abnormalities to improve or the effects of 16 weeks running therapy may be too subtle to impact (global) network communication of functional systems and may be limited to changes in localized brain regions. Discrepancies between our case-control results and that of previous literature are interpreted in light of methodological and clinical heterogeneity.

## Highlights

• We used data from The MOod Treatment with Antidepressants or Running (MOTAR) study.
• We studied the effects of running therapy on resting-state functional MRI measures.
• Running therapy improved clinical symptoms but had no effect on fMRI measures.
• Effects of running therapy may be too subtle to impact global functional communication.

## 1 Introduction

Depressive and anxiety disorders are prevalent and debilitating affective disorders that are associated with dysfunction of a multitude of different bodily systems, including the autonomic, immune, cardiovascular, metabolic and central nervous systems (Craske et al., 2017, Otte et al., 2016). Traditional treatments for these affective disorders consist of antidepressants (mainly selective serotonin re-uptake inhibitors [SSRI’s]) or psychotherapy (National Collaborating Centre for Mental Health, 2010, NICE, 2014). Although these treatment have shown to have roughly equal efficacy (Cuijpers et al., 2013), treatment resistance, relapses or adverse effects have urged clinical researchers to investigate alternative strategies, including exercise-based interventions such as running therapy. Aerobic exercise therapy has shown to be efficacious to prevent depression (Pearce et al., 2022) or improve clinically relevant symptoms of both depression and anxiety (Dauwan et al., 2019, Herring et al., 2012, Kandola et al., 2019, Keating et al., 2018, Miller et al., 2020, Netz, 2017) with an effect size comparable to that of pharmacological treatment (Blumenthal et al., 2007, Knapen et al., 2015). While recent *meta*-analyses favor a role for exercise therapy as augmentation strategy (Lee et al., 2021),it can also be considered as a standalone treatment for mild-moderate depression (Ravindran et al., 2016). This is especially relevant for individuals that are opposed to medication (González de León et al., 2022).

While exercise therapy has a positive influence on clinical symptoms, relatively little is known about the neurobiological mechanisms behind improving clinical symptoms by exercise. The MOod Treatment with Antidepressants or Running (MOTAR) study (https://www.motar.nl) was specifically designed to investigate the effects running therapy relative to antidepressants (SSRI’s) on biological outcomes, particularly physical health indicators, such as metabolic, immunological and autonomic markers (Lever-van Milligen et al., 2019). The results showed that although SSRI’s and running therapy had comparable favorable effects on remission of depressive and anxiety symptoms, running therapy showed a significant improvement in cardiovascular markers, weight, waist circumference and lung function compared with the SSRI group (Verhoeven et al., 2023). These significant differences were partly explained by a worsening of these physical health indicators in the SSRI group.

In the current study we investigated the effects of running therapy on functional network connectivity and topology as measured by resting-state functional magnetic resonance imaging (rs-fMRI) and graph analysis. In keeping with the triple-network theory (Menon, 2011), previous studies in individuals with a depressive or anxiety disorder have shown dysfunction of the default-mode network (DMN), salience network (SN) and frontoparietal network (FPN; also referred to as executive control network) (Brakowski et al., 2017, Kaiser et al., 2015, Kim and Yoon, 2018, Mulders et al., 2015, Northoff, 2020, Peterson et al., 2014, Picó-Pérez et al., 2017, Williams, 2017, Williams, 2016, Xu et al., 2019). Particularly the DMN and SN have been reported to show dysconnectivity both within and between networks, although the direction of the reported effect is heterogeneous (i.e. hyper- or hypoconnected) (Williams, 2017, Williams, 2016) and sub-modules of a network (e.g. anterior and posterior DMN) may be differentially affected (Doucet et al., 2020, Mulders et al., 2015). Most of this evidence is however based on seed-based connectivity studies that single out one (or a few) brain region(s). Graph-based analyses, however, better capture large-scale communication across the entire functional brain network and a *meta*-analysis showed significantly lower modularity and local efficiency in individuals with a depression compared with healthy controls (Xu et al., 2021). In this study we therefore used atlas-based graph analyses to examine case-control differences in network connectivity and topology using the pre-treatment scans of individuals with an affective disorder and matched healthy controls.

To the best of our knowledge, no study has yet investigated the effects of running therapy on functional connectivity in depressive and/or anxiety disorder individuals. Based on the few studies that investigated the effects of aerobic exercise training on functional connectivity in non-psychiatric populations, it seems that aerobic exercise particularly influences the DMN and associated brain areas, such as the hippocampal complex (Firth et al., 2018, Flodin et al., 2017, Li et al., 2017, McFadden et al., 2013, Porto et al., 2018, Tozzi et al., 2016). Some of these intervention studies also report effects on the sensorimotor network (Flodin et al., 2017), connectivity of the dlPFC (Prehn et al., 2019), and acute effects on amygdala reactivity during an emotional faces task (Chen et al., 2019).

This study investigates the short-term (immediately after treatment) and long-term effects (eight months after treatment) of running therapy on functional connectivity of the DMN and SN, and key brain regions known to be involved in the pathophysiology of affective disorders, i.e. the amygdala, hippocampus, sgACC and dlPFC. Because of its ability to provide a holistic view of large-scale communication across the entire network, we used a graph analytical approach, where we defined the entire brain as a network of nodes (brain areas) and edges (connections) and calculated connectivity strength and other topological measures (Rubinov and Sporns, 2010). We hypothesized that running therapy would normalize the network connectivity and topology of the aDMN, pDMN and SN and that these treatment-induced changes were related to symptom improvement. To infer the normalizing effects of running therapy we additionally performed a case-control analysis using the pre-treatment scans of individuals with an affective disorder and matched healthy controls. We also explored the effects of running therapy on modularity, the tendency of the whole-brain network to organize into densely connected communities (Rubinov and Sporns, 2010), and on clusters of edges that form an interconnected subnetwork using Network Based Statistics (NBS) (Zalesky et al., 2010).

## 2 Methods

### 2.1 Participants

Eligibility criteria for MOTAR are described in detail in the protocol paper (Lever-van Milligen et al., 2019). Briefly, individuals between 18–70 years and diagnosed with a current major depressive disorder, social phobia, generalized anxiety disorder, panic disorder or agoraphobia according to the DSM-IV criteria, were eligible for participation in this trial that recruited between July 2012 – July 2019. Diagnostic criteria were checked by a trained researcher according to the Composite International diagnostic interview (CIDI). Exclusion criteria were: use of antidepressants in the last two weeks, current use of other psychotropic medication apart from stable use of benzodiazepines, already exercising more than once a week, another psychiatric diagnosis other than depressive or anxiety disorder, acute suicidal risk, somatic contraindications to running therapy or treatment with antidepressant, pregnancy. Severe somatic contraindications that might interfere with safe participation in the running sessions were discussed with their own physician before enrollment. Healthy participants – without a history of any psychiatric disorder – were included as a comparison group to additionally perform case-control comparisons at baseline. For the MRI sub-study, participants additionally needed to be free from metallic objects (e.g. pacemakers) and not suffer from claustrophobia. All participants provided informed consent. The study was approved by the medical ethics committee of VU University medical center and conducted in accordance with the declaration of Helsinki. The trial was prospectively registered in the Netherlands Trial registry (NTR3460).

### 2.2 Study design

The MOTAR study had a partially randomized patient preference design where 141 individuals with depression and/or anxiety disorder were randomized or offered their preferred 16 week treatment of either: 1) treatment with the SSRI escitalopram at a dose of 10–20 mg daily (or sertraline 50–200 mg if escitalopram was poorly tolerated or ineffective) or 2) outdoor running therapy consisting of at least two sessions of 45 min per week. All participants were free from antidepressants or other psychotropic medication for at least two weeks prior to study participation, apart from stable (non-incidental) benzodiazepine use. Of the 141 individuals that participated in the MOTAR study (96 receiving running therapy, 45 antidepressants), 55 underwent rs-fMRI (40 running therapy; 15 SSRI group) (Verhoeven et al., 2023). Due to the small sample size of individuals with pre-post treatment imaging data in the SSRI group (N = 9), only the running therapy group is considered here, and no comparisons between groups were performed. Nevertheless, we report the effects of treatment on the full sample in the supplements. Each running session consisted of 10-min warming-up, 30-min jogging at 50–70 % of heart rate reserve during the first four weeks and 70–85 % during the subsequent 12 weeks and finished with a 5-min cooling down. Individuals were allowed to have one individual running session a week but were highly encouraged to participate in the three weekly organized group sessions. Treatment adherence was evaluated by attendance list during the group training sessions, supplemented by data from the heart rate monitors that participants wore during each running session. For more details on the interventions see (Lever-van Milligen et al., 2019). The intervention has previously been shown to be efficacious in alleviating depressive symptoms and was conducted using the Dutch evidence-based clinical guidelines (https://www.nhg.org/sites/default/files/content/nhg_org/uploads/multidisciplinaire_richtlijn_depressie_3e_revisie_2013.pdf). Clinical evaluation and MRI were administered before (baseline) and after the intervention (16 weeks) and follow-up (52 weeks). We additionally scanned 70 healthy controls.

### 2.3 Clinical measurements

At the day of MRI scanning we administered the Inventory of Depressive Symptomatology (IDS; 30 items) (Rush et al., 1996), the Beck Anxiety Inventory (BAI; 21 items) (Beck et al., 1961), and Fear Questionnaire (Marks and Mathews, 1979), to measure the severity of depressive, anxiety and phobia symptoms, respectively.

### 2.4 MRI acquisition

MRI scans were acquired at the Spinoza centre on a Philips 3 T Achieva MR (Philips, Best, the Netherlands) equipped with a 32-channel head coil. We acquired a 3D Turbo Field Echo (TFE) T1-weighted structural MRI with scan parameters: TR = 8.1 ms, TE = 3.7 ms, flip angle = 8 degrees, matrix size = 240 × 240, 1 mm^3^ isotropic voxels. The same scan protocols were used at all time points. Resting-state fMRI with eyes closed was acquired for 8 min with T2*-weighted echo-planar images (TR = 2300 ms, TE = 28 ms, flip angle = 76 degrees, 3.0 × 3.0 mm^2^ in plane resolution, 37 sequentially ascending slices of 3 mm with 0.3 mm gap, 210 volumes).

### 2.5 MRI processing

Structural MRI images were skull-stripped and segmented to reconstruct the brain surfaces using FreeSurfer 7.1.1. In case of longitudinal data (in individuals with an affective disorder only) we ran FreeSurfer on a mean robust template of the structural MRI scans of all available time points, otherwise we used the structural MRI at baseline. Resting-state fMRI images were preprocessed using fmriprep v20.2.1 (see supplementary methods for the boilerplate). Briefly, fMRI images from each time point were skull-stripped, realigned, slice-time corrected and corrected for susceptibility induced distortions using a ‘fieldmap-less’ approach. Noise regressors were extracted for further denoising the time-series. We simultaneously performed denoising and band pass filtering ([0.009–0.08 Hz]) using the denoiser tool (github.com/arielletambini/denoiser). We applied ‘ICAAROMA8Phys’ denoising that has previously been shown to provide a good denoising strategy in benchmark tests (Parkes et al., 2018). It consists of removing eight physiological signals from the white matter and cerebrospinal fluid (as well as their derivatives and quadratic terms) and the automatically identified motion-related components by automatic removal of motion artifacts using independent component analysis (ICA-AROMA) (Pruim et al., 2015). We additionally calculated the Framewise displacement (FD) and excluded all participants with a mean root mean squared FD > 0.5 mm or >20 volumes with >0.5 mm volume-to-volume displacement, indicative of excessive motion (Power et al., 2014, Power et al., 2012). Image quality metrics (e.g. DVARS and temporal SNR) were calculated using MRIqc (Esteban et al., 2017) and compared between groups and time-points.

Prior to timeseries extraction, the denoised functional images were mapped to the cortical surface. This approach has several advantages over a volume-based approaches including better adherence to cortical convolutions and better spatial localization (Coalson et al., 2018, Dickie et al., 2019). For this we used the ciftify tool that allows applying ‘human connectome project-style’ processing to legacy data (Dickie et al., 2019) and projection of the denoised functional volumes to the cortical surface and resampling to fsLR32K standard space. The resulting cortical surface images were merged with segmentation maps of the 14 subcortical areas (derived from FreeSurfer). See (Dickie et al., 2019) for more details. No smoothing was applied. Timeseries were extracted from 400 cortical brain areas that were parcellated according to the Schaefer atlas (Schaefer et al., 2018), and the 14 subcortical areas.

### 2.6 Connectivity measures

Timeseries of all 414 brain areas were cross-correlated using Pearson correlations to construct weighted, fully connected connectivity matrices; one for each participant at each timepoint. Each brain area was assigned to a functional network according to the Yeo’s 17 Network solution (Yeo et al., 2011). Next, we collapsed the 17 networks consisting of two visual, two somatomotor, two dorsal attention, two salience, two limbic, three frontoparietal and three default mode and one temporo-parietal network, into eight single networks of which we only considered the DMN and SN. The DMN was subsequently divided into an anterior (aDMN) and posterior (pDMN) part according to the spatial location of the node (see supplementary Table 1 for the node assignment) and the hippocampus was assigned to the pDMN. *Connectivity within networks* was defined as the average strength of all the nodes within a network and *connectivity between networks* as the overall average of the strength of the connections between two networks. We investigated case-control and therapy-induced differences in connectivity within and between the aDMN, pDMN and SN.

### 2.7 Topological measures

Topological measures were calculated using the brain connectivity toolbox (Rubinov and Sporns, 2010) on non-negative connectivity matrices because most network measures cannot be calculated with negative weights (we inverted the sign of these weights). We calculated the participation coefficient, within-module Z-degree and betweenness centrality of key regions in the pathophysiology of affective disorders to determine the role of these regions in long range network communication. These key regions were the amygdala, hippocampus, sgACC and DLPFC. *The participation coefficient* provides a measure for the connectivity of a node with its own module (i.e. community of tightly interconnected nodes) relative to other modules, while the *module Z-degree* relates to the node’s connectivity strength within its module only (Power et al., 2013). *Betweenness centrality* measures the influence of a node on global communication across the entire network (‘hub status’). We additionally explored the effects of running therapy on network measures of global communication across the entire brain network: *global efficiency* (ability of a network to exchange information), *modularity* (the degree to which the network can be divided into modules) and the *average participation coefficient*. Modularity was established by using a generalized Louvain method for community detection (Jeub et al., 2020). Robustness of the results were investigated by additionally using a wavelet coherence in the frequency range [0.009, 0.08 Hz] (Grinsted et al., 2004) to calculate the topological measures. This method and frequency range are less contaminated by head motion compared to Pearson correlation connectivity matrices (Mahadevan et al., 2020). Lastly, we explored the effects of running therapy on functional connectivity by applying network-based statistics (NBS) (Zalesky et al., 2010). NBS tests for potential statistically significant changes in edge strength for every cell in the connectivity matrix and identifies components based on clustering of these significant edges. For these clusters, permutation testing is applied to calculate P-values, where the actual clusters sizes are compared to a null distribution of maximum cluster sizes from random networks. Per recommendation of the developers in the reference manual (v1.2) we performed NBS over a range of thresholds (t = 1.41–4.24).

### 2.8 Data analyses

Case-control differences in clinical measures, image quality metrics, connectivity or topology were investigated using permutated Wilcoxon-Mann-Whitney tests using the coin package in R and we used Wilcoxon signed-rank tests to investigate the effects of running therapy. For both analyses, P-values were calculated based on 10,000 Monte Carlo resamples and a statistical threshold of P < 0.05. Results on the connectivity of the intrinsic networks (6 comparisons) or nodal level (8 comparisons) were corrected for multiple comparison using the False Discovery Rate (FDR; q < 0.05). The association between change in connectivity or topology and treatment-induced change in depressive (IDS) and anxiety symptom (BAI) severity were analyzed using repeated measures correlations (rmcorr package in R) (Bakdash and Marusich, 2017). Analyses were performed on the intention-to-treat sample but the robustness of the results were explored by additionally performing per-protocol analyses (taking into account compliance to therapy; defined as ≥22 session within 16 weeks (Verhoeven et al., 2023), based on evidence-based recommendations (Rethorst and Trivedi, 2013). We additionally explored the effects of running therapy at twelve month follow-up using a three time point repeated-measures ANOVA. This analysis plan was pre-registered: osf.io/986dj.

## 3 Results

### 3.1 Demographic and clinical characteristics

**Fig. 1:**
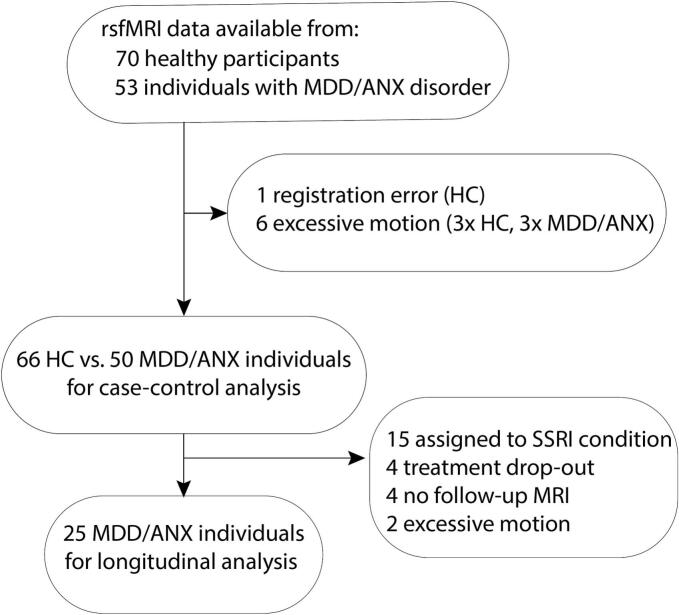
Flowchart. MDD/ANX = major depressive or anxiety disorder, HC = healthy control.

Nine participants had to be excluded, leaving 50 participants with a depressive or anxiety disorder and 66 healthy controls for analyses (see Fig. 1). An additional 25 individuals were excluded from the pre-to-post treatment analyses because they commenced SSRIs instead of running therapy (N = 15), showed motion artefacts (N = 2), did not receive a post-treatment MRI due to drop-out (N = 4) or refused to be rescanned (N = 4). Of the 50 individuals with an affective disorder, 20 % had major depressive disorder, 28 % had an anxiety disorder (social phobia, panic disorder or agoraphobia or generalized anxiety disorder) and 52 % had comorbid depression and anxiety disorder (see also Table 1). As expected, individuals with an affective disorder scored significantly higher on the IDS, BAI and FEAR questionnaires compared with healthy controls (all P’s < 0.001). Groups were well matched on age and sex, but not education (U = 1172.5, P = 0.009).

**Table 1:** Demographic and clinical characteristics.

	Healthy controls (n = 66)	Individuals with an affective disorder (N = 50)	Case-control Statistics	Running therapy sample (N = 25)^Ω^
Sex (N (%))				
Male	36 (54.5 %)	23 (46 %)	χ^2^_(1)_ = 0.83, P = 0.36	14 (66 %)
Female	30 (45.5 %)	27 (54 %)		11 (44 %)
Age (years) [range]	39.9 (14.04)	36.6 (11.8)	U = 1462, P = 0.30	40.2 (13.1) [22 – 66]
Education (years)	13.3 (3.3)	11.7 (3.1)	U = 1172.5P = 0.009	12.1 (2.8)
IDS	3.2 (2.5)	39.7 (13.9)	U = 0, P < 0.001	37.0 (13.8)
BAI	1.5 (2.0)	22.3 (12.2)	U = 23.5, P < 0.001	21.2 (11)
FEAR	8.8 (9.7)	40.9 (26.7)	U = 343, P < 0.001	34 (19.8)
Diagnosis^#^				
Major depressive disorder	−	36 (72 %)	−	16 (64 %)
Social Phobia	−	21 (42 %)	−	8 (32 %)
Panic disorder	−	21 (42 %)	−	7 (28 %)
Agoraphobia	−	13 (26 %)	−	5 (20 %)
Generalized Anxiety Disorder	−	15 (30 %)	−	9 (36)
Treatment (running therapy / SSRI)	−	35 / 15	−	25
Preferred or randomized treatment (%)	−	−	−	80 % / 20 %
Compliance*	−	−	−	57.1 %

Data are presented as mean (SD) unless otherwise indicated. # percentages do not add to 100 because patients may have multiple diagnoses. * Percentage of patients that followed the entire running therapy according to protocol. This includes the four (11.4 %) patients that dropped-out. Ω sample of N = 25 for which there was pre-treatment and post-treatment MRI available after applying exclusion criteria (see flowchart Fig. 1. *Abbreviations*: IDS = Inventory of depressive symptomatology; BAI = Beck Anxiety Inventory; FEAR = Fear Questionnaire, SSRI = selective serotonin reuptake inhibitors.

### 3.2 Baseline case-control analyses

Comparing healthy controls and individuals with an affective disorder at baseline revealed no differences in connectivity within or between the aDMN, pDMN and SN or differences in global or nodal topology (see Table 2). Similar results were obtained when using wavelet coherence as connectivity strength measure.

**Table 2:** Functional connectivity and topology analyses.

	Case-control		Running therapy		Case-control stats		Running therapy stats	
	Individuals with an affective disorder (N = 50) M ± SD	Healthy controls (N = 66) M ± SD	T0 (N = 25) M ± SD	T1 (N = 25) M ± SD	Z*	p-value_φ_	Z^#^	p-value_φ_
**Within network connectivity**								
aDMN	0.25 ± 0.07	0.25 ± 0.07	0.24 ± 0.07	0.23 ± 0.11	0.38	0.72	−0.39	0.71
pDMN	0.28 ± 0.06	0.25 ± 0.07	0.26 ± 0.05	0.25 ± 0.08	−1.45	0.15	−0.82	0.43
SN	0.32 ± 0.09	0.30 ± 0.09	0.34 ± 0.08	0.31 ± 0.10	−1.39	0.17	−0.98	0.33
**Between network connectivity**								
aDMN – pDMN	0.20 ± 0.06	0.20 ± 0.06	0.19 ± 0.05	0.19 ± 0.09	−0.15	0.89	0.04	0.73
aDMN – SN	0.09 ± 0.08	0.08 ± 0.07	0.09 ± 0.08	0.10 ± 0.10	−0.83	0.41	0.36	0.73
pDMN – SN	0.05 ± 0.08	0.04 ± 0.06	0.05 ± 0.09	0.05 ± 0.09	−0.28	0.77	0.12	0.92
**Global topology**								
GE	0.30 ± 0.03	0.30 ± 0.03	0.30 ± 0.03	0.30 ± 0.03	0.17	0.86	0.34	0.76
Q	0.11 ± 0.02	0.11 ± 0.02	0.11 ± 0.02	0.11 ± 0.02	0.35	0.72	0.61	0.57
Average PC	0.88 ± 0.02	0.88 ± 0.02	0.88 ± 0.02	0.88 ± 0.02	−0.41	0.69	−0.18	0.88
**Nodal topology (betweenness centrality** x 10^-3^**)**								
Amyg L	0.56 ± 0.65	0.66 ± 0.92	0.61 ± 0.69	0.62 ± 0.52	0.06	0.95	0.15	0.90
Amyg R	0.57 ± 0.77	0.54 ± 0.74	0.53 ± 0.65	0.38 ± 0.48	−0.25	0.81	−1.63	0.11
Hipp L	1.87 ± 1.81	1.64 ± 1.53	1.78 ± 1.71	1.88 ± 1.88	−0.50	0.62	0.09	0.94
Hipp R	1.87 ± 2.05	2.14 ± 2.10	1.82 ± 2.22	1.55 ± 0.99	1.19	0.23	0.58	0.58
DLPFC L	2.72 ± 1.91	2.94 ± 2.87	2.51 ± 1.65	2.54 ± 2.24	−0.38	0.71	−0.77	0.46
DLPFC R	2.26 ± 2.08	2.43 ± 2.14	2.10 ± 2.10	2.63 ± 2.45	0.66	0.50	0.09	0.93
sgACC L	0.44 ± 0.38	0.60 ± 0.62	0.44 ± 0.41	0.63 ± 1.0	1.07	0.28	0.85	0.41
sgACC R	0.56 ± 0.60	0.64 ± 0.78	0.64 ± 0.72	0.42 ± 0.37	−0.15	0.88	0.96	0.35

*Mann-Whitney *U* test with 10,000 resamples, # Wilcoxon signed rank test with 10,000 resamples. _φ_ P-values are *uncorrected* for False Discovery rate. All P_FDR_ > 0.05. On the nodal level only betweenness centrality values are reported. Participation coefficient and within Z degree showed similar results (see supplementary Table 2) Abbreviations:aDMN = anterior default mode network, pDMN = posterior default mode network, SN = Salience network, GE = global efficiency, Q = modularity, PC = participation coefficient, Amyg = Amygdala, Hipp = hippocampus, DLPFC = dorsolateral prefrontal cortex, sgACC = subgenual anterior cingulate cortex.

### 3.3 Post-treatment changes following 16-week running therapy

After running therapy, the 25 individuals with depression and/or anxiety disorder showed a significant decrease in scores on the IDS (Z = −4.13, P < 0.001), BAI (Z = -2.87, P = 0.003) and FEAR questionnaires (Z = -2.41, P = 0.015). Individuals (n = 10) that were assigned to running therapy but excluded from analyses (see Fig. 1) showed no differences in age, sex, education level or baseline IDS or BAI score, although their score on the FEAR questionnaire was significantly higher (U = 57, P = 0.017).

**Fig. 2:**
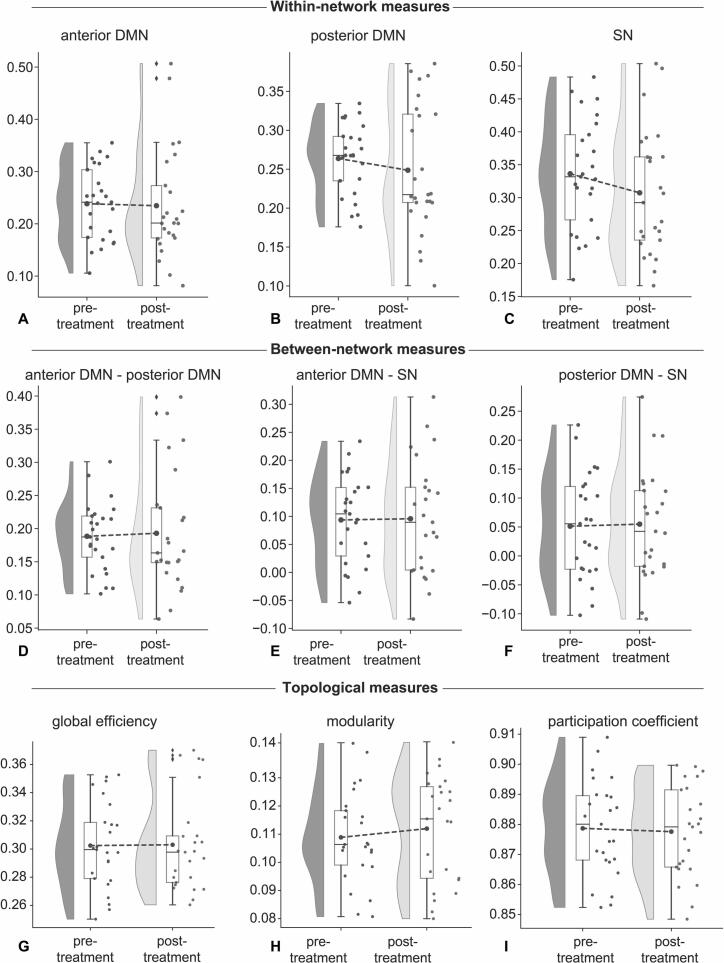
Effects of 16-week running therapy on within and between network connectivity and global topology in individuals with an affective disorder (n = 25). There were no running therapy induced changes on any of the within (A-C) or between (D-F) connectivity or topological (G-I) measures. Abbreviations: DMN = default mode network, SN = Salience Network, T0 = baseline assessment, T1 = assessment after 16-week running therapy.

Running therapy did not have a significant impact on the 16-week changes in within or between network connectivity, as well as on global or nodal topology (see Table 2, Fig. 2 and supplementary Table 2). This was observed when either using Pearson correlations or wavelet coherence as connectivity strength measure. In addition, we did not find any significant correlations between the improvements in clinical symptoms (as measured by the IDS, BAI or FEAR questionnaire) and changes in any of the connectivity measures over the 16-week period. Only considering individuals that were compliant to running therapy (N = 18; per protocol sample) did not change these results. Analyses on the full sample of individuals that completed either running therapy or SSRI treatment showed similar results (supplementary Table 4). Our exploratory analysis using NBS at multiple thresholds (t = 1.41–4.24) did not reveal statistically significant changes in edge strength following the 16-week period of running therapy. Results on the image quality measures are reported in the supplementary material and supplementary Fig. 2.

### 3.4 Exploratory long-term changes at one year follow-up

A total of 16 individuals completed the one-year follow-up assessment. Similar to the T0-T1 results, the longitudinal data for connectivity and global topological measures indicated no significant changes after running therapy (see supplementary figure 3 and supplementary Table 3).

### 3.5 Bayes factor

To follow-up on the non-significant case-control effects and effects of running therapy on functional network topology at 16 weeks, we calculated the Bayes Factor (BF) using the *BayesFactor R* package. BFs provide a direct comparison of the relative support for two competing theories (i.e. the null vs alternative hypothesis), based on the observed data. They provide several advantages over (post-hoc) power calculations, including offering a more comprehensive understanding of factors such as type II errors (Dienes, 2014). BFs are reported in Supplementary tables 4–5. While the strength of evidence was weak and inconclusive in some instances (0.33 < BF < 3), a consistent pattern emerged for the majority of our analyses concerning within and between network connectivity, as well as global topology when comparing cases to controls and assessing the impact of running therapy. In most cases, the BF was below or close to 0.33. This indicates strong to moderate support for the ‘evidence of absence’, effectively favoring the null hypothesis (Dienes, 2014, Keysers et al., 2020).

## 4 Discussion

In this study we investigated case-control differences and examined the effects of 16 weeks of running therapy on resting-state functional connectivity and network topology in individuals with a depressive or anxiety disorder. While we previously observed significant improvement of mental and physical health indicators (e.g., metabolic, immunological and autonomic markers) among the broader sample of 96 individuals (Verhoeven et al., 2023), our findings here indicate that running therapy did not induce alterations in brain network connectivity within a subsample of 25 individuals, despite significant improvements in depression and anxiety symptoms. No differences were observed between cases and controls at baseline. Post-hoc calculation of the Bayes factors tentatively suggests that these null findings are not merely a consequence of low statistical power. Image quality was also similar between time points, except for the temporal SNR which decreased over time.

Although multiple studies have shown beneficial effects of aerobic exercise on mood symptoms (Bailey et al., 2018, Stubbs et al., 2017), the neurobiological effects of exercise, including on neuroimaging markers, have been scarcely investigated (Schuch et al., 2016). Meta-analyses have consistently reported that exercise therapy increases hippocampal volume (Firth et al., 2018, Li et al., 2017), including in individuals with depression (Krogh et al., 2014), yet exercise-induced changes in functional connectivity are more heterogeneous and showed tentative increases in connectivity of local regions such as the hippocampus and cingulate cortex with other areas of the brain (Li et al., 2017). Others have observed normalization of local resting-state synchronous activity (Shen et al., 2024) and degree centrality (Huang et al., 2024) after exercise therapy in individuals with a subthreshold depression. Another study that investigated the effects of exercise on mood and functional connectivity showed that 16 weeks of physical exercise was able to improve mood in 46 healthy sedentary individuals accompanied by a reduction in local efficiency of the parahippocampal gyrus and higher connectivity of this area with supramarginal gyrus, superior temporal gyrus/pole and precentral area (Tozzi et al., 2016). To the best of our knowledge no such studies have been performed in individuals clinically diagnosed with a depression or anxiety disorder. The limited available evidence suggests that exercise may primarily exert regional effects on the brain, particularly on the morphometry of the hippocampus but additional studies are needed to verify this. Our analyses were focused on the impact of running therapy on the connectivity of *entire functional systems* such as the DMN, global (i.e. whole-brain) topology and the influence of specific nodes on brain-wide network communication. Despite symptom improvements, these relatively global analyses showed no effects of running therapy on network communication, and more localized effects of the intervention may have been averaged out due to our method of choice. The repeated measures correlation between the change in clinical symptoms after running therapy and change in network measures was also not significant, suggesting a relative disconnect between clinical behavior and communication of the currently studied brain networks. This is also exemplified by the fact that symptom severity was not associated with network measures at baseline (data not shown). Future studies with larger samples are needed to confirm these findings.

Our study also did not reveal significant differences between cases and controls, implying typically functioning brain networks in the patient group. Given this, it is perhaps not surprising that running therapy had little room to improve the functional connectivity and topology. At first glance, these findings contrast that of previous literature that generally showed dysconnectivity of the DMN and SN in individuals with an affective disorder, most notably MDD. However, the majority of previous investigations into functional connectivity relied on seed-based methodologies, which entail comparing the connectivity pattern of one particular brain region between groups (Kaiser et al., 2015, Xu et al., 2019). Alternatively, they have relied on independent component analysis (ICA), a data-driven but reproducible approach to identify “components” comprising brain voxels with synchronous activity which are thought to represent functional networks (Beckmann et al., 2005). With either method, the focus is primarily regional differences in functional connectivity as opposed to large-scale dysconnectivity of entire functional systems. A similar argument may apply here as for the absence of an effect of running therapy: the current atlas-based approach with graph measures may have overlooked regional or localized effects. Nevertheless, graph analysis offers several advantages over seed-based methods, including a more accurate description of the complex architecture of the entire human brain compared to seed-to-region correlations and calculation of measures that capture the fundamental principles of brain networks: integration and segregation (Rubinov and Sporns, 2010). A recent *meta*-analysis involving 12 rsfMRI graph studies showed that depressed individuals show significantly lower modularity and local efficiency compared to healthy controls (Xu et al., 2021). Both these findings point towards less segregation and higher coupling between functional systems. In the current study, we were unable to replicate these findings, as neither our modularity analyses nor our analyses of functional connectivity differences between functional systems yielded significant results. It is important to note, however, that sensitivity analyses showed that the *meta*-analytical results for modularity were mainly driven by a single study (Ma et al., 2020). Furthermore, these results also pertained to individuals receiving medication, while no differences between medication-free individuals and healthy controls were found. In our study, individuals were free from psychotropic medication apart from stable benzodiazepine use (Verhoeven et al., 2023). This discrepancy in medication use might account for the lack of significant findings in our sample. Interestingly, a multicenter mega-analysis conducted by Javaheripour and colleagues (2021) involving 606 depressed individuals and 476 controls, also did not observe any between-group differences in the connectivity within the DMN or of the DMN with other networks. However, they did observe lower DMN connectivity in medicated vs unmedicated individuals (Javaheripour et al., 2021). This further aligns with the results reported by Yan et al. (2019), where they found lower DMN connectivity only in (recurrent) individuals who were using antidepressants (Yan et al., 2019).

Javaheripour and colleagues also emphasize that previous reports on DMN connectivity patterns may be confounded by multiple factors, not only medication status and predominant reliance on seed-based analyses, but also depression subtype and severity as well as publication and confirmation bias. Alterations in the DMN (and FPN) may therefore not be considered a stable biomarker of MDD. A similar argument could be made for (comorbid) anxiety disorders. In fact, a recent study found no differences in functional connectivity of the SN or basal ganglia relative to healthy controls (Nawijn et al., 2022). Nevertheless, so far connectivity patterns in anxiety disorders have been relatively understudied. Further large-scale studies on functional connectivity alterations in affective disorders that use harmonized (pre)processing pipelines and take relevant covariates, most notably medication status and subtypes (e.g. immune-metabolic depression), into account. In this regard, the currently ongoing functional connectivity analyses by the Enhancing Neuro Imaging Genetics Through Meta Analysis (ENIGMA) MDD working group that involves several thousand participants may provide an important piece of that puzzle.

Strengths of this study are the fact what we pre-registered our analyses and that symptom improvement was also observed in this MRI subsample of the MOTAR study (Verhoeven et al., 2023). Limitations of this study are the relatively small MRI subsample that also precluded us from looking at the effects of running therapy on subtypes (e.g. the immunometabolic subtype) or specific affective disorders (e.g. MDD, generalized anxiety). It should, however, be noted that there also is high comorbidity between the disorders, both in our sample and in the general population. Larger sample sizes are needed to better dissect the clinical and biological heterogeneity of the disorders.

In conclusion, whereas running therapy had significant clinical benefits for individuals with an affective disorder, these benefits were not accompanied by changes in the connectivity or topology of the brain network, nor did we observe any case-control differences at baseline. We hypothesize that the effects of 16 weeks running therapy may be too subtle to alter the communication between entire functional systems and may instead instigate subtle local effects for which our network approach was not sufficiently sensitive.

## CRediT authorship contribution statement

**Chris Vriend:** Writing – review & editing, Writing – original draft, Visualization, Methodology, Formal analysis, Conceptualization. **Josine E. Verhoeven:** Writing – review & editing, Project administration, Investigation, Data curation. **Laura S. van Velzen:** Investigation. **Lianne Schmaal:** Writing – review & editing, Project administration, Investigation. **Brenda W.J.H. Penninx:** Writing – review & editing, Resources, Funding acquisition. **Laura K.M. Han:** Writing – review & editing, Project administration, Methodology, Investigation, Data curation.

## Declaration of competing interest

The authors declare that they have no known competing financial interests or personal relationships that could have appeared to influence the work reported in this paper.

## Data Availability

According to European law (GDPR) data containing potentially identifying or sensitive patient information are restricted; our data involving clinical participants are not freely available in a public repository. However, we highly value scientific collaboration, therefore, in principle, MOTAR data are available to scientific researchers working at non-commercial research organizations worldwide. Researchers can request existing data for data analyses.
