# Supplementary material for: Running therapy improves clinical symptoms but not functional network connectivity in individuals with affective disorders
Source: Neuroimage Clin. 2025 May 27;47:103812. doi: 10.1016/j.nicl.2025.103812 (PMC12166777; doi:10.1016/j.nicl.2025.103812)
Supplement: Supplementary Data 1 [file mmc1.docx]

SUPPLEMENTARY MATERIALS

**Contents**

- Fmriprep boilerplate
- Image quality measures
- Supplementary figures 1-3
- Supplementary Tables 1-5

**Fmriprep boilerplate**

Results included in this manuscript come from preprocessing performed using fMRIPrep 20.2.1 (Esteban, Markiewicz, et al. (2018); Esteban, Blair, et al. (2018); RRID:SCR_016216), which is based on Nipype 1.5.1 (Gorgolewski et al. (2011); Gorgolewski et al. (2018); RRID:SCR_002502).

Anatomical data preprocessing

A total of 3 T1-weighted (T1w) images were found within the input BIDS dataset. All of them were corrected for intensity non-uniformity (INU) with N4BiasFieldCorrection (Tustison et al. 2010), distributed with ANTs 2.3.3 (Avants et al. 2008, RRID:SCR_004757). The T1w-reference was then skull-stripped with a Nipype implementation of the antsBrainExtraction.sh workflow (from ANTs), using OASIS30ANTs as target template. Brain tissue segmentation of cerebrospinal fluid (CSF), white-matter (WM) and gray-matter (GM) was performed on the brain-extracted T1w using fast (FSL 5.0.9, RRID:SCR_002823, Zhang, Brady, and Smith 2001). A T1w-reference map was computed after registration of 3 T1w images (after INU-correction) using mri_robust_template (FreeSurfer 6.0.1, Reuter, Rosas, and Fischl 2010). Brain surfaces were reconstructed using recon-all (FreeSurfer 6.0.1, RRID:SCR_001847, Dale, Fischl, and Sereno 1999), and the brain mask estimated previously was refined with a custom variation of the method to reconcile ANTs-derived and FreeSurfer-derived segmentations of the cortical gray-matter of Mindboggle (RRID:SCR_002438, Klein et al. 2017). Volume-based spatial normalization to two standard spaces (MNI152NLin6Asym, MNI152NLin2009cAsym) was performed through nonlinear registration with antsRegistration (ANTs 2.3.3), using brain-extracted versions of both T1w reference and the T1w template. The following templates were selected for spatial normalization: FSL’s MNI ICBM 152 non-linear 6th Generation Asymmetric Average Brain Stereotaxic Registration Model [Evans et al. (2012), RRID:SCR_002823; TemplateFlow ID: MNI152NLin6Asym], ICBM 152 Nonlinear Asymmetrical template version 2009c [Fonov et al. (2009), RRID:SCR_008796; TemplateFlow ID: MNI152NLin2009cAsym],

Functional data preprocessing

For each of the 3 BOLD runs found per subject (across all tasks and sessions), the following preprocessing was performed. First, a reference volume and its skull-stripped version were generated using a custom methodology of fMRIPrep. A deformation field to correct for susceptibility distortions was estimated based on fMRIPrep’s fieldmap-less approach. The deformation field is that resulting from co-registering the BOLD reference to the same-subject T1w-reference with its intensity inverted (Wang et al. 2017; Huntenburg 2014). Registration is performed with antsRegistration (ANTs 2.3.3), and the process regularized by constraining deformation to be nonzero only along the phase-encoding direction, and modulated with an average fieldmap template (Treiber et al. 2016). Based on the estimated susceptibility distortion, a corrected EPI (echo-planar imaging) reference was calculated for a more accurate co-registration with the anatomical reference. The BOLD reference was then co-registered to the T1w reference using bbregister (FreeSurfer) which implements boundary-based registration (Greve and Fischl 2009). Co-registration was configured with six degrees of freedom. Head-motion parameters with respect to the BOLD reference (transformation matrices, and six corresponding rotation and translation parameters) are estimated before any spatiotemporal filtering using mcflirt (FSL 5.0.9, Jenkinson et al. 2002). BOLD runs were slice-time corrected using 3dTshift from AFNI 20160207 (Cox and Hyde 1997, RRID:SCR_005927). The BOLD time-series were resampled onto the following surfaces (FreeSurfer reconstruction nomenclature): fsnative, fsaverage5. The BOLD time-series (including slice-timing correction when applied) were resampled onto their original, native space by applying a single, composite transform to correct for head-motion and susceptibility distortions. These resampled BOLD time-series will be referred to as preprocessed BOLD in original space, or just preprocessed BOLD. The BOLD time-series were resampled into standard space, generating a preprocessed BOLD run in MNI152NLin6Asym space. First, a reference volume and its skull-stripped version were generated using a custom methodology of fMRIPrep. Automatic removal of motion artifacts using independent component analysis (ICA-AROMA, Pruim et al. 2015) was performed on the preprocessed BOLD on MNI space time-series after removal of non-steady state volumes and spatial smoothing with an isotropic, Gaussian kernel of 6mm FWHM (full-width half-maximum). Corresponding “non-aggresively” denoised runs were produced after such smoothing. Additionally, the “aggressive” noise-regressors were collected and placed in the corresponding confounds file. Several confounding time-series were calculated based on the preprocessed BOLD: framewise displacement (FD), DVARS and three region-wise global signals. FD was computed using two formulations following Power (absolute sum of relative motions, Power et al. (2014)) and Jenkinson (relative root mean square displacement between affines, Jenkinson et al. (2002)). FD and DVARS are calculated for each functional run, both using their implementations in Nipype (following the definitions by Power et al. 2014). The three global signals are extracted within the CSF, the WM, and the whole-brain masks. Additionally, a set of physiological regressors were extracted to allow for component-based noise correction (CompCor, Behzadi et al. 2007). Principal components are estimated after high-pass filtering the preprocessed BOLD time-series (using a discrete cosine filter with 128s cut-off) for the two CompCor variants: temporal (tCompCor) and anatomical (aCompCor). tCompCor components are then calculated from the top 2% variable voxels within the brain mask. For aCompCor, three probabilistic masks (CSF, WM and combined CSF+WM) are generated in anatomical space. The implementation differs from that of Behzadi et al. in that instead of eroding the masks by 2 pixels on BOLD space, the aCompCor masks are subtracted a mask of pixels that likely contain a volume fraction of GM. This mask is obtained by dilating a GM mask extracted from the FreeSurfer’s aseg segmentation, and it ensures components are not extracted from voxels containing a minimal fraction of GM. Finally, these masks are resampled into BOLD space and binarized by thresholding at 0.99 (as in the original implementation). Components are also calculated separately within the WM and CSF masks. For each CompCor decomposition, the k components with the largest singular values are retained, such that the retained components’ time series are sufficient to explain 50 percent of variance across the nuisance mask (CSF, WM, combined, or temporal). The remaining components are dropped from consideration. The head-motion estimates calculated in the correction step were also placed within the corresponding confounds file. The confound time series derived from head motion estimates and global signals were expanded with the inclusion of temporal derivatives and quadratic terms for each (Satterthwaite et al. 2013). Frames that exceeded a threshold of 0.5 mm FD or 1.5 standardised DVARS were annotated as motion outliers. All resamplings can be performed with a single interpolation step by composing all the pertinent transformations (i.e. head-motion transform matrices, susceptibility distortion correction when available, and co-registrations to anatomical and output spaces). Gridded (volumetric) resamplings were performed using antsApplyTransforms (ANTs), configured with Lanczos interpolation to minimize the smoothing effects of other kernels (Lanczos 1964). Non-gridded (surface) resamplings were performed using mri_vol2surf (FreeSurfer).

Many internal operations of fMRIPrep use Nilearn 0.6.2 (Abraham et al. 2014, RRID:SCR_001362), mostly within the functional processing workflow. For more details of the pipeline, see the section corresponding to workflows in fMRIPrep’s documentation.

Copyright Waiver

The above boilerplate text was automatically generated by fMRIPrep with the express intention that users should copy and paste this text into their manuscripts unchanged. It is released under the CC0 license.

References

Abraham, Alexandre, Fabian Pedregosa, Michael Eickenberg, Philippe Gervais, Andreas Mueller, Jean Kossaifi, Alexandre Gramfort, Bertrand Thirion, and Gael Varoquaux. 2014. “Machine Learning for Neuroimaging with Scikit-Learn.” Frontiers in Neuroinformatics 8. https://doi.org/10.3389/fninf.2014.00014.

Avants, B.B., C.L. Epstein, M. Grossman, and J.C. Gee. 2008. “Symmetric Diffeomorphic Image Registration with Cross-Correlation: Evaluating Automated Labeling of Elderly and Neurodegenerative Brain.” Medical Image Analysis 12 (1): 26–41. https://doi.org/10.1016/j.media.2007.06.004.

Behzadi, Yashar, Khaled Restom, Joy Liau, and Thomas T. Liu. 2007. “A Component Based Noise Correction Method (CompCor) for BOLD and Perfusion Based fMRI.” NeuroImage 37 (1): 90–101. https://doi.org/10.1016/j.neuroimage.2007.04.042.

Cox, Robert W., and James S. Hyde. 1997. “Software Tools for Analysis and Visualization of fMRI Data.” NMR in Biomedicine 10 (4-5): 171–78. https://doi.org/10.1002/(SICI)1099-1492(199706/08)10:4/5<171::AID-NBM453>3.0.CO;2-L.

Dale, Anders M., Bruce Fischl, and Martin I. Sereno. 1999. “Cortical Surface-Based Analysis: I. Segmentation and Surface Reconstruction.” NeuroImage 9 (2): 179–94. https://doi.org/10.1006/nimg.1998.0395.

Esteban, Oscar, Ross Blair, Christopher J. Markiewicz, Shoshana L. Berleant, Craig Moodie, Feilong Ma, Ayse Ilkay Isik, et al. 2018. “FMRIPrep.” Software. Zenodo. https://doi.org/10.5281/zenodo.852659.

Esteban, Oscar, Christopher Markiewicz, Ross W Blair, Craig Moodie, Ayse Ilkay Isik, Asier Erramuzpe Aliaga, James Kent, et al. 2018. “fMRIPrep: A Robust Preprocessing Pipeline for Functional MRI.” Nature Methods. https://doi.org/10.1038/s41592-018-0235-4.

Evans, AC, AL Janke, DL Collins, and S Baillet. 2012. “Brain Templates and Atlases.” NeuroImage 62 (2): 911–22. https://doi.org/10.1016/j.neuroimage.2012.01.024.

Fonov, VS, AC Evans, RC McKinstry, CR Almli, and DL Collins. 2009. “Unbiased Nonlinear Average Age-Appropriate Brain Templates from Birth to Adulthood.” NeuroImage 47, Supplement 1: S102. https://doi.org/10.1016/S1053-8119(09)70884-5.

Gorgolewski, K., C. D. Burns, C. Madison, D. Clark, Y. O. Halchenko, M. L. Waskom, and S. Ghosh. 2011. “Nipype: A Flexible, Lightweight and Extensible Neuroimaging Data Processing Framework in Python.” Frontiers in Neuroinformatics 5: 13. https://doi.org/10.3389/fninf.2011.00013.

Gorgolewski, Krzysztof J., Oscar Esteban, Christopher J. Markiewicz, Erik Ziegler, David Gage Ellis, Michael Philipp Notter, Dorota Jarecka, et al. 2018. “Nipype.” Software. Zenodo. https://doi.org/10.5281/zenodo.596855.

Greve, Douglas N, and Bruce Fischl. 2009. “Accurate and Robust Brain Image Alignment Using Boundary-Based Registration.” NeuroImage 48 (1): 63–72. https://doi.org/10.1016/j.neuroimage.2009.06.060.

Huntenburg, Julia M. 2014. “Evaluating Nonlinear Coregistration of BOLD EPI and T1w Images.” Master’s Thesis, Berlin: Freie Universität. http://hdl.handle.net/11858/00-001M-0000-002B-1CB5-A.

Jenkinson, Mark, Peter Bannister, Michael Brady, and Stephen Smith. 2002. “Improved Optimization for the Robust and Accurate Linear Registration and Motion Correction of Brain Images.” NeuroImage 17 (2): 825–41. https://doi.org/10.1006/nimg.2002.1132.

Klein, Arno, Satrajit S. Ghosh, Forrest S. Bao, Joachim Giard, Yrjö Häme, Eliezer Stavsky, Noah Lee, et al. 2017. “Mindboggling Morphometry of Human Brains.” PLOS Computational Biology 13 (2): e1005350. https://doi.org/10.1371/journal.pcbi.1005350.

Lanczos, C. 1964. “Evaluation of Noisy Data.” Journal of the Society for Industrial and Applied Mathematics Series B Numerical Analysis 1 (1): 76–85. https://doi.org/10.1137/0701007.

Power, Jonathan D., Anish Mitra, Timothy O. Laumann, Abraham Z. Snyder, Bradley L. Schlaggar, and Steven E. Petersen. 2014. “Methods to Detect, Characterize, and Remove Motion Artifact in Resting State fMRI.” NeuroImage 84 (Supplement C): 320–41. https://doi.org/10.1016/j.neuroimage.2013.08.048.

Pruim, Raimon H. R., Maarten Mennes, Daan van Rooij, Alberto Llera, Jan K. Buitelaar, and Christian F. Beckmann. 2015. “ICA-AROMA: A Robust ICA-Based Strategy for Removing Motion Artifacts from fMRI Data.” NeuroImage 112 (Supplement C): 267–77. https://doi.org/10.1016/j.neuroimage.2015.02.064.

Reuter, Martin, Herminia Diana Rosas, and Bruce Fischl. 2010. “Highly Accurate Inverse Consistent Registration: A Robust Approach.” NeuroImage 53 (4): 1181–96. https://doi.org/10.1016/j.neuroimage.2010.07.020.

Satterthwaite, Theodore D., Mark A. Elliott, Raphael T. Gerraty, Kosha Ruparel, James Loughead, Monica E. Calkins, Simon B. Eickhoff, et al. 2013. “An improved framework for confound regression and filtering for control of motion artifact in the preprocessing of resting-state functional connectivity data.” NeuroImage 64 (1): 240–56. https://doi.org/10.1016/j.neuroimage.2012.08.052.

Treiber, Jeffrey Mark, Nathan S. White, Tyler Christian Steed, Hauke Bartsch, Dominic Holland, Nikdokht Farid, Carrie R. McDonald, Bob S. Carter, Anders Martin Dale, and Clark C. Chen. 2016. “Characterization and Correction of Geometric Distortions in 814 Diffusion Weighted Images.” PLOS ONE 11 (3): e0152472. https://doi.org/10.1371/journal.pone.0152472.

Tustison, N. J., B. B. Avants, P. A. Cook, Y. Zheng, A. Egan, P. A. Yushkevich, and J. C. Gee. 2010. “N4ITK: Improved N3 Bias Correction.” IEEE Transactions on Medical Imaging 29 (6): 1310–20. https://doi.org/10.1109/TMI.2010.2046908.

Wang, Sijia, Daniel J. Peterson, J. C. Gatenby, Wenbin Li, Thomas J. Grabowski, and Tara M. Madhyastha. 2017. “Evaluation of Field Map and Nonlinear Registration Methods for Correction of Susceptibility Artifacts in Diffusion MRI.” Frontiers in Neuroinformatics 11. https://doi.org/10.3389/fninf.2017.00017.

Zhang, Y., M. Brady, and S. Smith. 2001. “Segmentation of Brain MR Images Through a Hidden Markov Random Field Model and the Expectation-Maximization Algorithm.” IEEE Transactions on Medical Imaging 20 (1): 45–57. https://doi.org/10.1109/42.906424.

**Image quality measures**

we performed a case-control and longitudinal analyses of the image quality measures (IQMs) derived from MRIqc: Entropy-focus criterion (EFC) provides a measure for the amount of ghosting or motion-related blurring of the scan, full-width half maximum smoothness is indicative of the overall blurriness of the scan, DVARS measures the mean rate of change of voxel intensities across the entire scan duration, while the framewise displacement (FD) provides an index of how much frame-to-frame displacement a participant showed by averaging translation and rotation parameters. Lastly, the temporal signal-to-noise ratio (tSNR) provides an approximation of the quality of the timeseries by dividing the mean whole-brain voxel intensities across time by the standard deviation. See mriqc.readthedocs.io/en/latest/measures.html and Esteban et al. 2017 for more details about these IQMs.

There were no differences in EFC, FWHM smoothness, DVARS or FD between mood disorder patients and healthy controls at baseline. Temporal signal to noise ratio was however significantly higher in patients compared with healthy controls (Z=-3.56, P<0.001; supplementary figure 1). Over time, scans of individuals with an affective disorder showed a significant decrease in tSNR (Z=-4.29, P<0.001) but not the other IQMs.


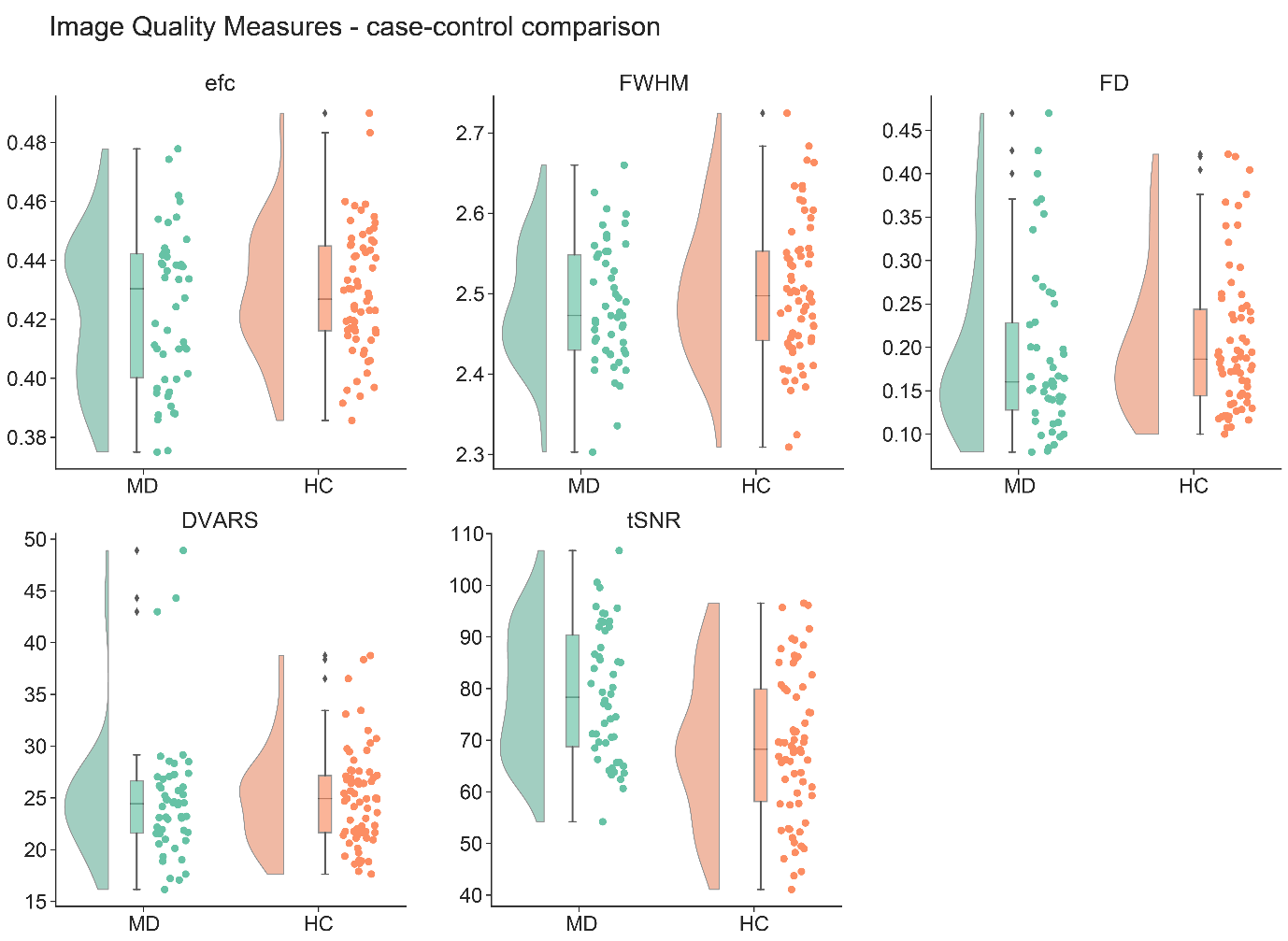


**Supplementary Figure 1 – Image quality measures of depressive and anxiety disorder patient and healthy controls at baseline.** Abbreviations: EFC: entropy-focus criterion, FWHM: full-width half maximum, tSNR: temporal signal-to-noise ratio, FD: framewise displacement, DVARS: spatial standard deviation of successive difference images, MD: individuals with a depressive or anxiety disorder, HC: healthy controls.


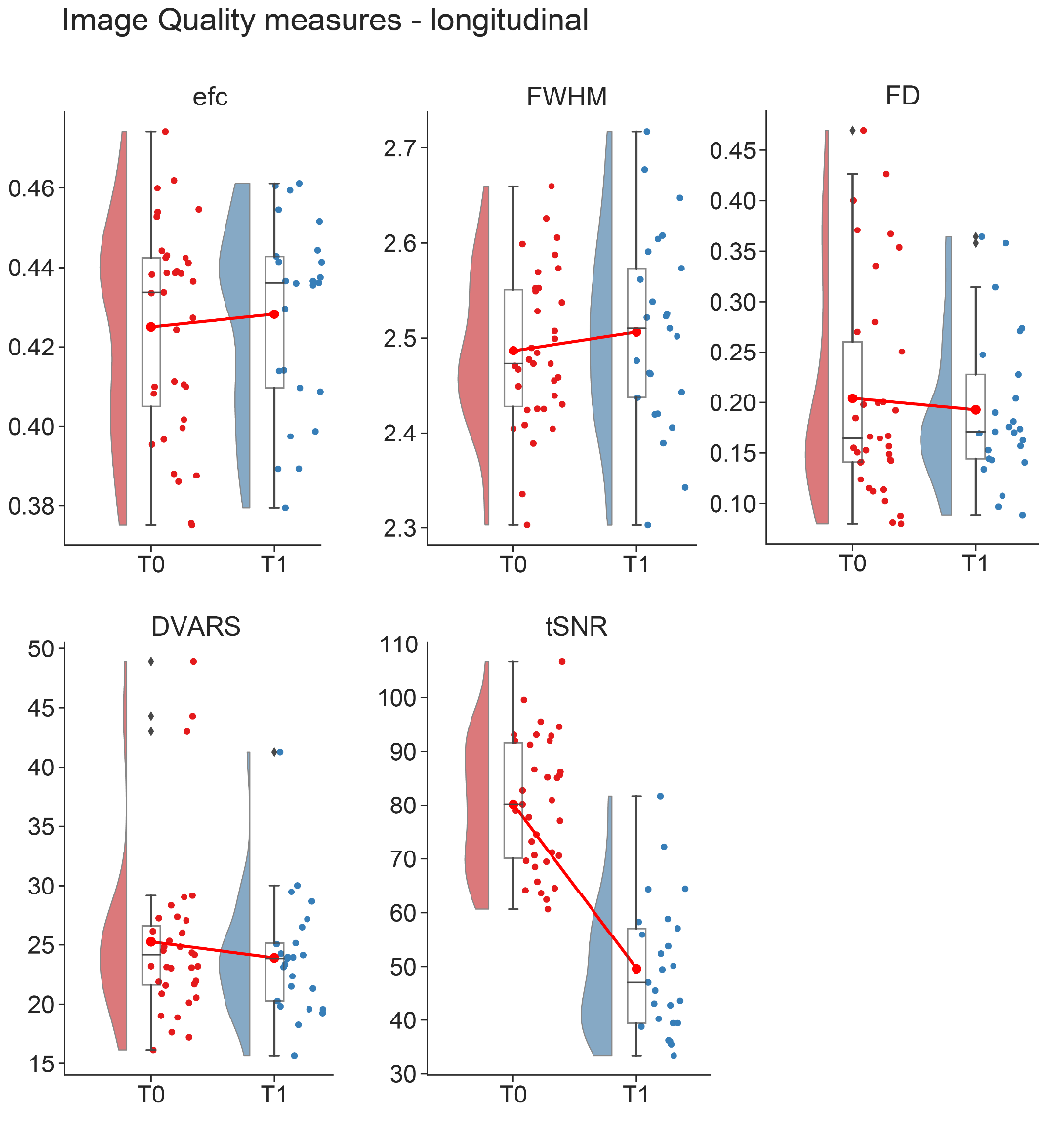


**Supplementary Figure 2 – Image quality measures of individuals with a depressive and anxiety disorder before (T0) and after (T1) running therapy.**


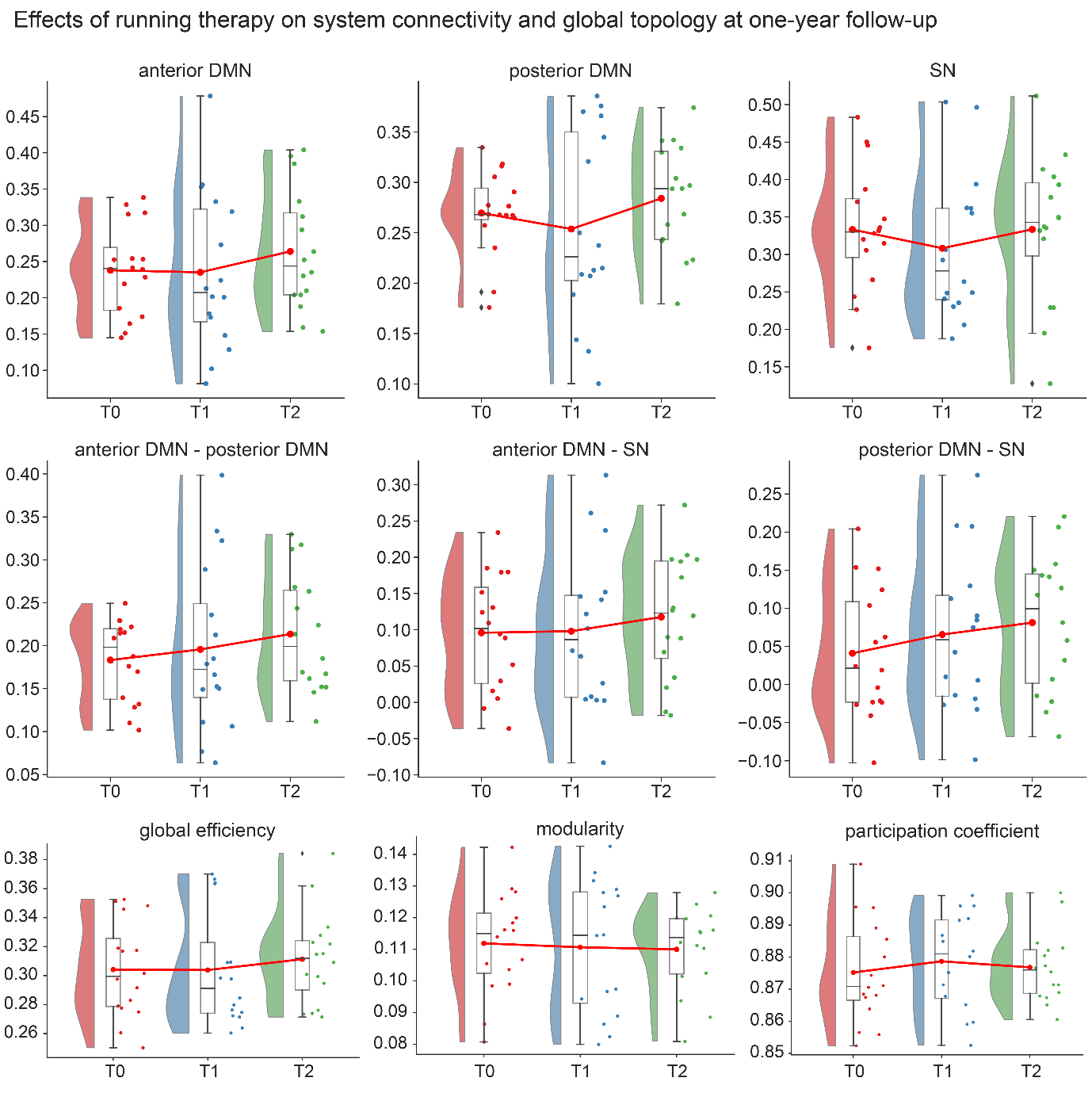
 **Supplementary Figure 3 – effects of running therapy on within and between functional network connectivity and network topology.** Abbreviations: DMN = default mode network, SN = Salience Network, T0 = baseline assessment, T1 = assessment after 16-week running therapy, T2= 52 weeks after running therapy.

| Supplementary Table 1 – node assignmnet DMN | |
| --- | --- |
| Schaefer 400 Node | DMN |
| 17Networks_LH_DefaultA_IPL_1 | anterior |
| 17Networks_LH_DefaultA_IPL_2 | anterior |
| 17Networks_LH_DefaultA_PFCd_1 | anterior |
| 17Networks_LH_DefaultA_PFCd_2 | anterior |
| 17Networks_LH_DefaultA_PFCd_3 | anterior |
| 17Networks_LH_DefaultA_PFCm_1 | anterior |
| 17Networks_LH_DefaultA_PFCm_2 | anterior |
| 17Networks_LH_DefaultA_PFCm_3 | anterior |
| 17Networks_LH_DefaultA_PFCm_4 | anterior |
| 17Networks_LH_DefaultA_PFCm_5 | anterior |
| 17Networks_LH_DefaultA_PFCm_6 | anterior |
| 17Networks_LH_DefaultB_Temp_1 | anterior |
| 17Networks_LH_DefaultB_PFCd_1 | anterior |
| 17Networks_LH_DefaultB_PFCd_2 | anterior |
| 17Networks_LH_DefaultB_PFCd_3 | anterior |
| 17Networks_LH_DefaultB_PFCd_4 | anterior |
| 17Networks_LH_DefaultB_PFCd_5 | anterior |
| 17Networks_LH_DefaultB_PFCd_6 | anterior |
| 17Networks_LH_DefaultB_PFCl_1 | anterior |
| 17Networks_LH_DefaultB_PFCl_2 | anterior |
| 17Networks_LH_DefaultB_PFCv_1 | anterior |
| 17Networks_LH_DefaultB_PFCv_2 | anterior |
| 17Networks_LH_DefaultB_PFCv_3 | anterior |
| 17Networks_LH_DefaultB_PFCv_4 | anterior |
| 17Networks_LH_DefaultB_PFCv_5 | anterior |
| 17Networks_RH_DefaultA_Temp_1 | anterior |
| 17Networks_RH_DefaultA_IPL_1 | anterior |
| 17Networks_RH_DefaultA_IPL_2 | anterior |
| 17Networks_RH_DefaultA_PFCd_1 | anterior |
| 17Networks_RH_DefaultA_PFCd_2 | anterior |
| 17Networks_RH_DefaultA_PFCm_1 | anterior |
| 17Networks_RH_DefaultA_PFCm_2 | anterior |
| 17Networks_RH_DefaultA_PFCm_3 | anterior |
| 17Networks_RH_DefaultA_PFCm_4 | anterior |
| 17Networks_RH_DefaultA_PFCm_5 | anterior |
| 17Networks_RH_DefaultA_PFCm_6 | anterior |
| 17Networks_RH_DefaultB_AntTemp_1 | anterior |
| 17Networks_RH_DefaultB_PFCd_1 | anterior |
| 17Networks_RH_DefaultB_PFCd_2 | anterior |
| 17Networks_RH_DefaultB_PFCd_3 | anterior |
| 17Networks_RH_DefaultB_PFCd_4 | anterior |
| 17Networks_RH_DefaultB_PFCd_5 | anterior |
| 17Networks_RH_DefaultB_PFCv_1 | anterior |
| 17Networks_RH_DefaultB_PFCv_2 | anterior |
| 17Networks_RH_DefaultB_PFCv_3 | anterior |
| 17Networks_LH_DefaultA_pCunPCC_1 | posterior |
| 17Networks_LH_DefaultA_pCunPCC_2 | posterior |
| 17Networks_LH_DefaultA_pCunPCC_3 | posterior |
| 17Networks_LH_DefaultA_pCunPCC_4 | posterior |
| 17Networks_LH_DefaultA_pCunPCC_5 | posterior |
| 17Networks_LH_DefaultA_pCunPCC_6 | posterior |
| 17Networks_LH_DefaultA_pCunPCC_7 | posterior |
| 17Networks_LH_DefaultB_Temp_2 | posterior |
| 17Networks_LH_DefaultB_Temp_3 | posterior |
| 17Networks_LH_DefaultB_Temp_4 | posterior |
| 17Networks_LH_DefaultB_Temp_5 | posterior |
| 17Networks_LH_DefaultB_Temp_6 | posterior |
| 17Networks_LH_DefaultB_IPL_1 | posterior |
| 17Networks_LH_DefaultB_IPL_2 | posterior |
| 17Networks_LH_DefaultC_IPL_1 | posterior |
| 17Networks_LH_DefaultC_Rsp_1 | posterior |
| 17Networks_LH_DefaultC_Rsp_2 | posterior |
| 17Networks_LH_DefaultC_Rsp_3 | posterior |
| 17Networks_LH_DefaultC_PHC_1 | posterior |
| 17Networks_LH_DefaultC_PHC_2 | posterior |
| 17Networks_LH_DefaultC_PHC_3 | posterior |
| 17Networks_RH_DefaultA_pCunPCC_1 | posterior |
| 17Networks_RH_DefaultA_pCunPCC_2 | posterior |
| 17Networks_RH_DefaultA_pCunPCC_3 | posterior |
| 17Networks_RH_DefaultA_pCunPCC_4 | posterior |
| 17Networks_RH_DefaultA_pCunPCC_5 | posterior |
| 17Networks_RH_DefaultB_Temp_1 | posterior |
| 17Networks_RH_DefaultB_Temp_2 | posterior |
| 17Networks_RH_DefaultC_IPL_1 | posterior |
| 17Networks_RH_DefaultC_IPL_2 | posterior |
| 17Networks_RH_DefaultC_Rsp_1 | posterior |
| 17Networks_RH_DefaultC_Rsp_2 | posterior |
| 17Networks_RH_DefaultC_PHC_1 | posterior |
| 17Networks_RH_DefaultC_PHC_2 | posterior |

| **Supplementary Table 2 – nodal topology analyses** | | | | |
| --- | --- | --- | --- | --- |
|  | **Case-control stats** | | **Running therapy stats** | |
|  | Z^*^ | p-valueᵩ | Z^#^ | p-valueᵩ |
| **Participation coefficient** | | | | |
| Amyg L | -1.137 | 0.256 | 0.067 | 0.955 |
| Amyg R | 2.124 | 0.034 | 0.578 | 0.578 |
| Hipp L | 0.97 | 0.334 | 0.659 | 0.521 |
| Hipp R | 1.07 | 0.289 | 1.762 | 0.08 |
| DLPFC L | 0.19 | 0.847 | -0.767 | 0.46 |
| DLPFC R | -0.619 | 0.544 | 0.094 | 0.934 |
| sgACC L | -1.199 | 0.234 | 0.848 | 0.413 |
| sgACC R | 0.195 | 0.844 | 0.955 | 0.353 |
| **Within Z-degree** | | | | |
| Amyg L | 0.212 | 0.840 | -0.202 | 0.846 |
| Amyg R | -0.797 | 0.432 | -0.309 | 0.773 |
| Hipp L | -1.433 | 0.152 | -1.251 | 0.219 |
| Hipp R | -0.797 | 0.413 | -0.874 | 0.392 |
| DLPFC L | -0.413 | 0.680 | -0.928 | 0.368 |
| DLPFC R | -0.251 | 0.671 | -0.094 | 0.935 |
| sgACC L | 1.628 | 0.103 | 0.256 | 0.81 |
| sgACC R | -0.251 | 0.804 | -1.547 | 0.122 |
| *Mann-Whitney U test with 10,000 resamples, # Wilcoxon signed rank test with 10,000 resamples. ᵩ P-values are *uncorrected* for False Discovery rate. All P_FDR_ > 0.05. Abbreviations: Amyg = Amygdala, Hipp = hippocampus, DLFPFC = dorsolateral prefrontal cortex, sgACC = subgenual anterior cingulate cortex | | | | |

| **Supplementary Table 3 – Functional connectivity and topology analyses at one year follow-up** | | |
| --- | --- | --- |
|  | F(2,30)-Statistic* | P-value |
| **Within network connectivity** | | |
| aDMN | 0.81 | 0.45 |
| pDMN | 1.06 | 0.36 |
| SN | 0.64 | 0.54 |
| **Between network connectivity** | | |
| aDMN – pDMN | 0.79 | 0.47 |
| aDMN – SN | 0.77 | 0.47 |
| pDMN – SN | 1.82 | 0.18 |
| **Global topology** | | |
| GE | 0.34 | 0.72 |
| Q | 0.06 | 0.94 |
| Average PC | 0.38 | 0.55 |
| Abbreviations: aDMN = anterior default mode network, pDMN = posterior default mode network, SN = Salience network, GE = global efficiency, Q = modularity, PC = participation coefficient. *three time point repeated-measures ANOVA | | |

| **Supplementary Table 4 – changes in functional connectivity and topology of running therapy and SSRI sample combined** | | | |
| --- | --- | --- | --- |
|  | **Pre-post stats (N=34)** | | |
|  | Z^#^ | p-valueᵩ | BF |
| **Within network connectivity** | | | |
| aDMN | -0.385 | 0.715 | 0.18 |
| pDMN | -1.393 | 0.173 | 0.47 |
| SN | -1.376 | 0.172 | 0.64 |
| **Between network connectivity** | | | |
| aDMN – pDMN | -0.231 | 0.831 | 0.18 |
| aDMN – SN | -0.111 | 0.918 | 0.19 |
| pDMN – SN | -0.658 | 0.527 | 0.21 |
| **Global topology** | | | |
| GE | -0.556 | 0.587 | 0.23 |
| Q | 0.47 | 0.649 | 0.19 |
| Average PC | 0.282 | 0.796 | 0.19 |
| **Nodal topology (betweenness centrality)** | | | |
| Amyg L | 0.504 | 0.625 | 0.20 |
| Amyg R | -0.898 | 0.38 | 0.19 |
| Hipp L | -0.299 | 0.777 | 0.18 |
| Hipp R | 1.325 | 0.197 | 0.18 |
| DLPFC L | 0.333 | 0.748 | 0.20 |
| DLPFC R | 0.778 | 0.444 | 0.22 |
| sgACC L | -0.128 | 0.903 | 0.38 |
| sgACC R | -0.701 | 0.489 | 0.41 |
| **Nodal topology (Participation coefficient)** | | | |
| Amyg L | -0.128 | 0.901 | 0.19 |
| Amyg R | 0.915 | 0.368 | 0.36 |
| Hipp L | 0.47 | 0.648 | 0.25 |
| Hipp R | 1.53 | 0.127 | 0.74 |
| DLPFC L | -0.333 | 0.755 | 0.18 |
| DLPFC R | 0.265 | 0.804 | 0.18 |
| sgACC L | 0.949 | 0.346 | 0.35 |
| sgACC R | 1.12 | 0.27 | 0.48 |
| **Nodal topology (Within Z-degree)** | | | |
| Amyg L | 0.402 | 0.697 | 0.20 |
| Amyg R | -0.402 | 0.699 | 0.19 |
| Hipp L | -0.556 | 0.59 | 0.25 |
| Hipp R | -0.504 | 0.625 | 0.21 |
| DLPFC L | -1.428 | 0.158 | 0.53 |
| DLPFC R | -0.727 | 0.473 | 0.21 |
| sgACC L | 0.214 | 0.843 | 0.19 |
| sgACC R | -1.787 | 0.075 | 1.1 |
| Wilcoxon signed rank test with 10,000 resamples. ᵩ P-values are *uncorrected* for False Discovery rate. All P_FDR_ > 0.05. Abbreviations: Amyg = Amygdala, Hipp = hippocampus, DLFPFC = dorsolateral prefrontal cortex, sgACC = subgenual anterior cingulate cortex | | | |

| **Supplementary table 5 – Bayes factors of the functional connectivity and topology analyses** | | |
| --- | --- | --- |
|  | **Case-control Bayes factor** | **Running therapy Bayes factor** |
| **Within network connectivity** | | |
| aDMN | 0.22 | 0.21 |
| pDMN | 0.93 | 0.29 |
| SN | 0.34 | 0.44 |
| **Between network connectivity** | | |
| aDMN – pDMN | 0.22 | 0.21 |
| aDMN – SN | 0.28 | 0.21 |
| pDMN – SN | 0.21 | 0.21 |
| **Global topology** | | |
| GE | 0.20 | 0.21 |
| Q | 0.20 | 0.25 |
| Average PC | 0.21 | 0.22 |
| **Nodal topology (betweenness centrality** x 10^-3^**)** | | |
| Amyg L | 0.46 | 0.21 |
| Amyg R | 0.23 | 0.36 |
| Hipp L | 0.58 | 0.21 |
| Hipp R | 0.27 | 0.24 |
| DLPFC L | 0.39 | 0.21 |
| DLPFC R | 0.23 | 0.34 |
| sgACC L | 0.21 | 0.34 |
| sgACC R | 0.20 | 1.01 |

REFERENCES

Esteban O, Birman D, Schaer M, Koyejo OO, Poldrack RA, Gorgolewski KJ. MRIQC: Advancing the automatic prediction of image quality in MRI from unseen sites. PLoS One. 2017;12(9):e0184661.
